# MethylomeMiner: A novel tool for high-resolution analysis of bacterial methylation patterns from nanopore sequencing

**DOI:** 10.1016/j.csbj.2025.10.047

**Published:** 2025-10-24

**Authors:** Marketa Jakubickova, Katerina Sabatova, Michaela Zbudilova, Matej Bezdicek, Martina Lengerova, Helena Vitkova

**Affiliations:** a Department of Biomedical Engineering, Brno University of Technology, Technicka 12, Brno, 61200, Czechia; b Division of Clinical Microbiology and Immunology, Department of Laboratory Medicine, University Hospital Brno and Faculty of Medicine of Masaryk University, Jihlavska 20, Brno, 62500, Czechia

**Keywords:** Methylome, Epigenetics, BedMethyl tables, Pangenome, Python package

## Abstract

DNA methylation plays a key role in gene regulation, genome stability, bacterial adaptation, and many other essential cellular processes. Thanks to nanopore sequencing technology, it is now possible to detect these modifications during sequencing without any prior chemical treatment. However, methylation data processing and their interpretation in a biological context remain challenging as there are no convenient and easy-to-use tools available for this purpose. Therefore, here, we present a simple Python-based tool, MethylomeMiner, to process methylation calls from nanopore sequencing. The tool allows high-confidence methylation sites to be selected based on coverage and methylation rate and assigned to coding or non-coding regions using genome annotation. In addition, the tool supports population-level analysis using pangenome data to compare methylation patterns across multiple bacterial genomes. Altogether, MethylomeMiner provides a straightforward and reproducible workflow that can be easily integrated into existing analyses and helps uncover the functional roles of DNA methylation in bacterial genomes.

## Graphical Abstract

**Figure:**
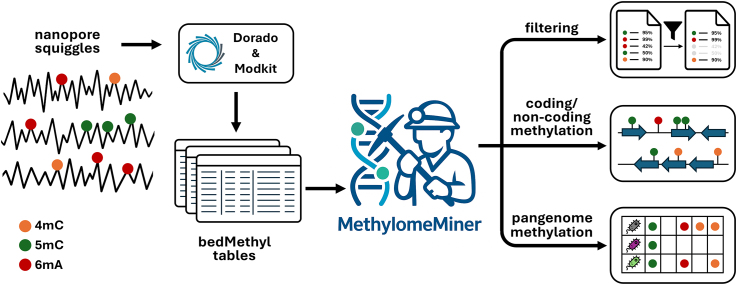

## 1 Introduction

DNA methylation, a fundamental epigenetic modification that involves adding a methyl group to nucleotides, plays a key role in regulating gene expression, maintaining genome stability, and mediating cellular adaptability [1]. Over the last few years, it has been extensively studied in eukaryotes, especially in humans, where it influences processes such as development, disease progression, and cell differentiation [2], [3]. On the contrary, methylation roles and mechanisms in bacterial genomes are less explored despite their growing recognition as key players in various cellular processes. Bacteria exhibit three main types of DNA methylation: N4-methylcytosine (4mC), C5-methylcytosine (5mC), and N6-methyladenine (6mA), which regulate crucial processes including DNA replication and repair, virulence factor expression, environmental adaptation, and host-pathogen interactions [4], [5]. Therefore, further research is needed to better understand their specific functions and roles in bacterial systems.

Recent advances in sequencing technologies have significantly changed the study of bacterial methylomes. In particular, Oxford Nanopore Technologies (ONT) has enabled direct DNA methylation detection without the need for DNA chemical treatment or modification, as was the case with bisulfite sequencing. Nanopore sequencing identifies methylation by detecting alterations in the electrical current generated as the DNA strand passes through the pore located in the membrane [6]. The methylation information is therefore embedded in the raw signal, allowing it to be detected during basecalling. Nowadays, it is possible to detect, besides commonly studied methylation types (4mC, 5mC and 6mA), other types such as 5-hydroxymethylcytosine (5hmC), 5-methylcytosine at CpG sites (5mCG) and 5-hydroxymethylcytosine at CpG sites (5hmCG) [7].

Over the past decade, numerous tools have been developed to directly detect DNA methylation from raw nanopore sequencing signals. One of the earliest such tools was Nanopolish, which enables the detection of 5mC in human methylome data [8]. This was followed by tools such as Tombo, which supports the identification of 5mC, 6mA, CpG methylation, and even *de novo* methylation events [9]; SignalAlign, which employs probabilistic signal alignment for modification detection [10]; and mCaller, specifically designed for 6mA detection [11]. Subsequently, additional tools emerged, including NanoMod, which enables single-base resolution detection for DNA modifications [12], DeepMod, designed for genome-wide DNA modification identification using deep learning [13], and Nanodisco, which facilitates *de novo* methylation typing and DNA methylation pattern fine-mapping [14]. However, except for the recently introduced DeepMod2 [15], most of these tools were designed for nanopore data generated using the older ONT R9 chemistry and are thus incompatible with the current R10 version.

With the introduction of the new ONT basecaller Dorado (https://github.com/nanoporetech/dorado), released alongside the R10 chemistry, methylation detection can now be performed directly during basecalling using a single, integrated tool. In combination with ONT’s Modkit utility, methylation information can be converted from BAM files to bedMethyl files, an already well-established format for methylation data in the context of bisulfite sequencing data. However, the resulting files are typically large and contain vast amounts of raw information, making efficient downstream processing and interpretation a significant challenge. Despite these advances, there is currently a lack of specialized software tools capable of rapid, streamlined analysis of such data, particularly in the context of bacterial methylomes.

Although several existing tools offer valuable methylation analysis functionalities, most are either tailored to specific tasks or primarily optimized for eukaryotic data. For instance, MIJAMP and Nanoident support motif discovery and methylation type classification [16], [17], while diffONT focuses on predicting methylation-specific PCR primer regions [18]. The pycoMeth tool enables methylome segmentation and differential methylation analysis, but is predominantly designed for human data [19]. In the context of bacterial methylation analysis, the aforementioned Modkit (https://github.com/nanoporetech/modkit) offers basic functionality for differential methylation analysis by comparing sample pairs at the regional or single-nucleotide level. However, a major limitation of this approach is the requirement that all reads must be aligned to a single reference sequence. As a result, only genomic regions shared across all samples can be analysed [20], which is particularly impractical when analysing bacterial strains that exhibit substantial variability in genome content.

To address these challenges, we introduce a novel Python-based package, MethylomeMiner, designed specifically to analyze bacterial methylation data in bedMethyl format generated by Dorado and Modkit. MethylomeMiner provides streamlined preprocessing, filtering, and statistical analysis of methylation datasets, and integrates genomic annotations to identify methylation patterns in both coding and non-coding regions. Furthermore, it supports population-level comparisons using pangenome data, enabling cross-strain methylation profiling and facilitating more profound insights into the functional roles of DNA methylation in genome regulation, environmental adaptation, and pathogenicity.

## 2 Implementation

**Fig. 1:**
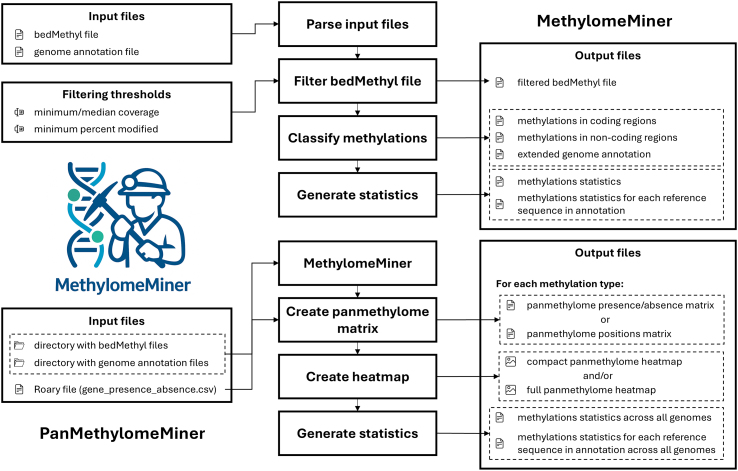
MethylomeMiner flowchart shows functional blocks of MethylomeMiner and PanMethylomeMiner together with their inputs and outputs.

MethylomeMiner consists of two functional modules with separate command-line interfaces (see Fig. 1): (i) MethylomeMiner - to filter bedMethyl files and classify methylation sites with respect to genomic features, and (ii) PanMethylomeMiner - to integrate methylation data across the bacterial pangenome. The software is available at https://github.com/BioSys-BUT/MethylomeMiner with a detailed user guide.

For MethylomeMiner development, Python (v3.12) was used together with several external packages. Parsing raw bedMethyl files was performed by pandas (v2.2.3, [21]) with pyarrow (18.1.0) engine, and pandas DataFrame data structure was utilized to manipulate data. Genome annotation files were parsed according to their format, either by biopython (v1.84, [22]) for GenBank format or bcbio-gff (v0.7.1) for GFF v3 format. Graphical outputs were generated with seaborn (v0.13.2, [23]), SciPy (v1.16.1, [24]) and fastcluster (v1.3.0, [25]) libraries. Finally, the Click (v8.1) package was employed to build a command-line interface.

### 2.1 MethylomeMiner: Filtering and classification of methylation data

In the first part, bedMethyl files containing methylation data derived from nanopore sequencing by Dorado and Modkit are preprocessed and filtered. This step involves verifying the input file structure to ensure they are correctly formatted, i.e., checking the number of columns and validating the data types in each column. If the structure is valid, the bedMethyl files are parsed, and the methylation type codes are renamed for better readability and subsequent analysis. Next, the identified modified nucleotides listed in bedMethyl files are filtered based on coverage and methylation rate. The coverage threshold is determined using the “score” column in the bedMethyl file, which represents the total valid coverage and is calculated as the sum of the number of reads with modified bases, the number of reads with unmodified (canonical) bases, and the number of reads with modifications different from the specified one. The user can either set the minimum coverage threshold based on their own data characteristics (e.g., sequencing depth and quality, single-genome vs. population data) or provide a path to a directory with more bedMethyl files, and the threshold will be determined by first calculating the median coverage for each bedMethyl input file and then taking the median of those values across all files. The methylation rate is derived from the "percent_modified" column, which represents the percentage of reads supporting methylation, calculated as the number of reads with modified bases divided by the valid coverage. The default minimum methylation rate is 90 %, but the user can adjust it to any value between 0 % and 100 %, depending on how strict the filtering should be. Thus, in population studies, a more relaxed threshold can be used to retain more sites for comparison, while in single-genome analyses, a stricter threshold can be applied to focus on only high-confidence sites. After filtering, the table showing methylated nucleotide positions can be saved in JSON, CSV, TSV or in the original bedMethyl file format.

The second part of the tool analyzes methylation patterns in the analyzed genome’s coding and non-coding regions. For this purpose, the user must provide a genome annotation file in either GFF v3 or GenBank format, which is then processed to extract information about coding sequences (CDS), including gene IDs, start and end positions of CDS, the strands on which genes are located, and gene products. The filtered methylation data from the previous step are then combined with the annotation data to identify whether the methylation sites are in coding or non-coding regions. Moreover, for methylation sites located in non-coding regions, the tool identifies the genes situated upstream and downstream of methylated bases. This information can help analyze the potential methylation regulatory roles in promoter regions or other intergenic elements. As a result of this part, two CSV files are generated for each analyzed bedMethyl table: one containing information about methylations in coding regions, including positions and the types of methylated bases within genes, and the other containing methylations in non-coding regions with neighboring genes’ annotations and their respective positions. Additionally, an extended genome annotation CSV file is created, where each CDS feature has a list of found base modification positions distinguished by methylation type. Finally, general filtered methylation count statistics are provided in a CSV file as a table that summarizes the filtered methylated base counts, showing the number of methylation sites detected in coding and non-coding regions, along with their totals and the overall number of methylations. Users can also request to save the outputs separately for each reference sequence in the annotation, depending on their analysis requirements.

### 2.2 PanMethylomeMiner: Pangenome-wide methylation integration

Moreover, the tool focuses on analyzing methylation sites within the pangenome. The user must provide output files from the Roary pangenome analysis tool to perform this analysis. Specifically, the required inputs include the gene_presence_absence.csv file and corrected GFF files, in which the locus tags for individual CDS have been renamed to match the gene names used in the Roary output. The tool processes these inputs to create extended genome annotations with information on methylation in the coding sequences by MethylomeMiner. Then, for each genome, the tool finds the pangenome genes in the extended annotation using the gene_presence_absence.csv table and for each methylation type (e.g., 4mC, 5mC, and 6mA) creates a matrix where rows are the genes from the pangenome and columns are the analyzed genomes. The values in the matrix can be, based on users’ choice, binary to indicate the presence/absence of methylations (0 for no detected methylation, 1 for some detected methylation), or a list of genomic positions where methylations were identified within pangenome genes. In both cases, if the gene is not found in the genome, no value is assigned. The resulting matrices are saved as separate CSV files for each methylation type, along with a summary table of methylation counts (similar to MethylomeMiner statistics) across all analyzed genomes.

In the PanMethylomeMiner module, an optional function is implemented to visualize core/pangenome methylation patterns using clustered heatmaps. This function takes the binary matrix indicating the methylation presence/absence for individual methylation types and generates heatmaps showing methylation profiles across analyzed samples in PNG, SVG or PDF file format. By default, genes found in at least 95 % of the analyzed samples are evaluated, but the user can adjust this threshold. The matrix is clustered using hierarchical clustering based on Hamming distances, with average linkage used to group genes and genomes that exhibit similar methylation patterns. As a result, two heatmaps can be generated. The first is a high-resolution version with gene names displayed on the y-axis, suitable for a detailed study of specific genes. The second is a simplified, lower-resolution version without gene labels, designed for observing overall methylation trends. Methylation visualization using heatmaps supports the identification of consistently methylated core genes, strain- or lineage-specific methylation events, and genes with variable methylation across the population.

## 3 Use case: Methylation analysis of *K. pneumoniae*

The proposed package was tested on a dataset of 20 *Klebsiella pneumoniae* genomes to demonstrate its usability and illustrate the complete analysis workflow. The following section describes the generation and processing, including the preparation of inputs required for MethylomeMiner. The subsequent section presents the interpretation of the methylation results obtained by the tool. All data used and produced in the following section are available on Zenodo [26].

### 3.1 Data preparation workflow

20 *K. pneumoniae* genomes belonging to six sequence types (see Supplementary Table S1) were mainly collected from patients at the University Hospital Brno. DNA extraction was performed using DNeasy PowerSoil Pro Kit (Qiagen, Venlo, NL), and the DNA purity was subsequently assessed using a NanoDrop spectrophotometer (Thermo Fisher Scientific, Waltham, MA, USA). DNA concentration and fragment length were evaluated using the Qubit 3.0 Fluorometer (Thermo Fisher Scientific, Wilmington, DE, USA) and the Agilent 4200 TapeStation system (Agilent Technologies, Santa Clara, CA, USA), respectively. The sequencing library was prepared using Rapid Barcoding Kit 96 V14 (Oxford Nanopore Technologies, Oxford, UK) and sequenced on the PromethION 2 Solo sequencing platform (Oxford Nanopore Technologies, Oxford, UK) with R10.4.1 flow cells.

The obtained sequencing data were basecalled by Dorado integrated in MinKNOW (v23.11.5, https://nanoporetech.com/community) using the super-accurate model (dna_r10.4.1_e8.2_400bps_sup@v5.0.0), and the minimum read quality was set to 10. The obtained pass reads in fastq format were further processed. Firstly, reads were trimmed using Dorado (v0.7.3, https://github.com/nanoporetech/dorado), and secondly, they were mapped to the human genome (GCF_000001405.40) by minimap2 (v2.28, [27]) to remove possible contamination. Unmapped reads were extracted by Samtools (v1.10, [28]) and *de novo* assembled by Flye (v2.9.3, [29]).

Then, the obtained genomes were annotated. For consistent results, it is necessary to use the same annotation tool across all samples, as different tools yield different results. In our case, for each genome, the resulting contigs were annotated using DFAST (v1.3.1, [30]), which is widely used for bacterial genomes. Also, the chromosomes were simultaneously rearranged so that the *dnaA* gene would be the first. The obtained annotation files were used for pangenome analysis performed by Roary (v3.13.0, [31]) with the default settings. In the following step, the number of genes that formed the pangenome (all genes present in the analyzed dataset), core genome (genes present in all strains), accessory genomes (genes present in more than one strain but not in all), and unique genes (genes present in only one strain) were determined.

The obtained sequencing data were basecalled again in the same way (super-accurate model, Q ≥ 10) but with modified base detection, specifically 4mC, 5mC and 6mA. Then, using Dorado aligner, basecalled reads with detected modified bases were mapped to the corresponding *de novo* assembled rearranged genome (described above). Samtools was employed to sort and index the obtained assemblies, and finally, Modkit (v0.2.6, https://github.com/nanoporetech/modkit) with default settings was used to convert BAM files to bedMethyl tables, which were further processed and analyzed by the proposed tool.

### 3.2 Dataset summary

Sequencing of 20 *K. pneumoniae* genomes generated between 96,703 and 237,536 reads per genome, with an average of 173,436 reads. After removing reads with a quality of less than 10, trimming and removing human contamination, the average number of reads per genome decreased to 166,481. The average read length ranged from 2498 bp to 4766 bp per genome, with an average of 3190 bp. Detailed statistics for each sample are provided in Supplementary Table S2.

After genome assembly, one chromosome and from one to three plasmids were obtained for each analyzed genome. The mean sequencing coverage was 145×. The chromosome lengths varied from 5.19 Mb to 5.45 Mb, and the plasmid lengths ranged from 11.5 kbp to 268.6 kbp (see Supplementary Table S3). The average GC content was 57.1 %. The annotation revealed that the number of coding sequences (CDSs) ranged from 5156 to 5616, and there were 25 rRNAs, while the number of tRNAs varied from 85 to 89. Complete results can be seen in Supplementary Table S4.

For 20 *K. pneumoniae* genomes, the pangenome comprised 9088 genes, of which 4025 formed the core genome. In total, 2898 genes were identified in two or more genomes but not in all; thus, they were classified as a part of the accessory genome. 2165 genes were found in only one sample at a time; therefore, they were labelled as unique.

### 3.3 Methylation analysis using MethylomeMiner

#### 3.3.1 Filtering and processing of detected methylation sites

For each sample, the bedMethyl tables generated by the process described above were processed using the first MethylomeMiner module. Firstly, the tables needed to be filtered as Modkit attempted to assess the methylation status of each nucleotide in the genome, where methylation can occur (in our case, cytosine and adenine). This means that even in cases where none of the mapped reads show any sign of methylation at a given position, the site is still included in the output and reported with a methylation rate of zero. The filtration was based on two thresholds: the coverage of the methylated base and the methylation rate. The minimal coverage value was set to 36, corresponding to the median coverage across all analyzed bedMethyl tables. The minimum rate for a base to be considered methylated was set to 90 %. These thresholds were chosen with respect to our dataset to maintain a sufficient number of highly probable methylation sites while minimising false positives. The change in the number of reported methylations under different threshold settings is illustrated in Supplementary Fig. S1.

Across all genomes, a total of 1231,195 methylation sites were identified, comprising 2559 4mC (0.2 %), 447,999 5mC (36.4 %), and 780,637 6mA (63.4 %) modifications (see Supplementary Table S5), confirming that 6mA is the most prevalent methylation type in bacteria [32]. As the 6mA modification is involved in regulating virulence, stress response and antibiotic resistance [33], it was expected to be the dominant modification in the pathogenic *Klebsiella* species on which the package is tested.

On chromosomes, between 955 and 95,264 methylation sites were identified, with an average of 59,041, and their methylation type distribution was consistent with that observed across all genomes. On plasmids, the number of methylation sites ranged from 57 to 3366 (see Supplementary Table S6); however, since the length of plasmids is variable, unlike the length of chromosomes, the length of a particular plasmid influences the number of methylation sites.

#### 3.3.2 Methylation distribution in coding and non-coding regions

Using the MethylomeMiner module, the methylations with high confidence were classified according to their genomic context, distinguishing between coding and non-coding regions. Across all samples, a higher proportion of methylations was located in non-coding regions, specifically 55 % of 4mC, 54 % of 5mC, and 52 % of 6mA (see Supplementary Table S5). On average, one in every 473 nucleotides was methylated in coding regions, and one in every 56 nucleotides in non-coding regions. As non-coding regions form about 1 % of the genome, their methylation density is relatively high compared to that of coding regions.

While non-coding regions were densely methylated, coding regions generally contained only a small number of sites per gene, typically one to three for 5mC, one to six for 6mA, and never more than one for 4mC.

However, a small subset of genes showed a very high methylation load, with dozens of modified positions. For 5mC, more than 50 methylation sites were consistently found in *group_82*, a hypothetical protein on the chromosome, with the highest loads in KP1267 (61 sites), KP1829 (57), KP1821 (56), KP1834 (55), KP1822 (53), and KP1833 (51). In addition, one genome (KP1622) showed hypermethylation in *group_85*, another hypothetical protein, but located on a plasmid (60 sites). For 6mA, the same two loci were hypermethylated. The hypothetical protein *group_82* showed a high number of methylation sites in KP1833 (58), KP1822 (57), KP1821 (56), KP1829 (55), and KP1267 (52). In KP1622, hypermethylation was again detected in the hypothetical protein *group_85* with 53 sites. All genes remained below the 50-site threshold for 4mC modifications.

Further analysis using the BLAST protein (BLASTp) web tool (v2.17, [34]) revealed that both *group_82* and *group_85* show homology with phage-related proteins, specifically with TipJ proteins of the phage tail family. This suggests a possible phage origin or phage function for these loci, which is not surprising, as prophages or their remnants are often found in *K. pneumoniae* [35], [36].

#### 3.3.3 Pangenome methylation analysis

The second module, PanMethylomeMiner, was used to analyze methylation profiles across the bacterial pangenome, enabling genomes with similar overall methylation patterns to be identified, as well as genes that share similar methylation across the analyzed population.

For each analyzed methylation type, corresponding high- and low-resolution heatmaps were generated. The heatmaps show the presence/absence of methylation across genes present in at least 95 % of analyzed genomes, which results in a total of 4239 genes. The clustering shown in the heatmap groups genomes and genes with similar methylation profiles, enabling the identification of strain-level epigenetic similarities and genes consistently or variably methylated across the population.

**Fig. 2:**
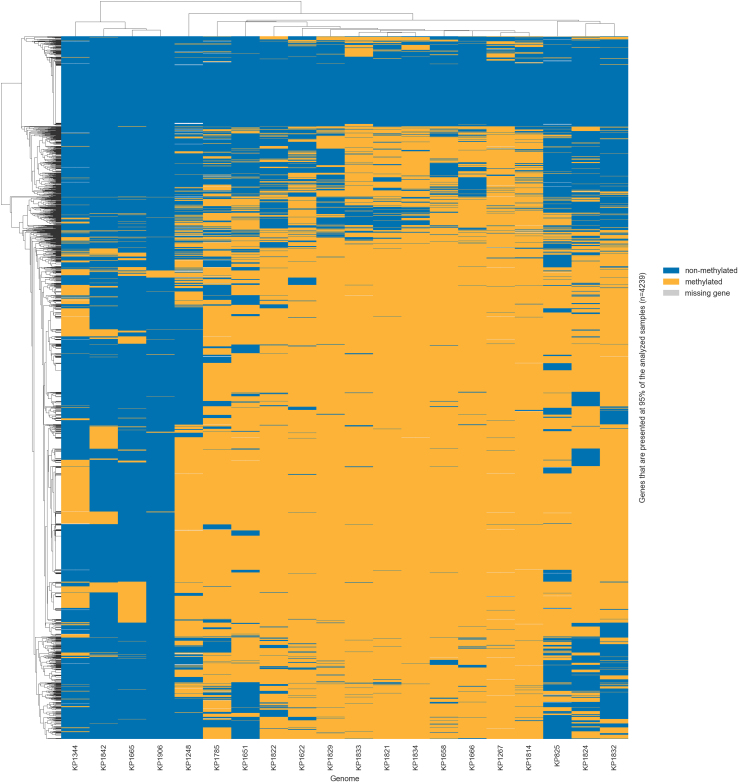
Heatmap showing 5mC methylation across 4239 core genes in 20 *K. pneumoniae* genomes. Only genes present in at least 95 % of genomes were included. Yellow indicates methylated genes, blue non-methylated genes, and grey genes not present in the genome. Rows (genes) and columns (genomes) are clustered based on methylation profile similarity. (For interpretation of the references to colour in this figure legend, the reader is referred to the web version of this article.)

In the case of 5mC and 6mA methylation, two main genome clusters can be observed, as can be seen in Fig. 2 and Supplementary Fig. S2, respectively. These clusters indicate that the genomes within them share similar methylation profiles. However, they do not correspond to the same sequence types, in contrast to previous observations [37], in which genomes clustered by sequence type, but there were large distances between individual strains within a cluster. This difference may be because the current analysis focuses only on core genes (present in at least 95 % of genomes) for comparison, so strain-specific genes or mobile genetic elements may be excluded. In contrast, no clear clustering was observed for 4mC methylation (see Supplementary Fig. S3), which is probably caused by the low number of 4mC methylations that occurred in the analyzed dataset. High-resolution heatmaps for all methylation types can be found at [26].

At the gene level, genes methylated in 95 % of strains were identified. In the case of 5mC methylation, 54 such genes were found, including one, *traI*, which was methylated in all analyzed samples. This gene encodes a bifunctional protein that is essential for conjugative DNA transfer [38]. For 6mA methylation, 87 genes were methylated in at least 95 % of samples. Among them, three genes were methylated in all strains: *traI*, *nirB* encoding the large subunit of nitrite reductase that reduces nitrite to ammonium for nitrogen assimilation [39]; and a gene encoding molybdopterin guanine dinucleotide-containing S/N-oxide reductase, an enzyme involved in anaerobic respiration by reducing sulfur- and nitrogen-oxide compounds [40]. In contrast, no genes methylated in ≥ 95 % of strains were identified for 4mC, which reflects this modification’s very low abundance in the dataset.

### 3.4 Methylation consistency validation

The methylation detection consistency was assessed by adding one isolate of *K. pneumoniae* (KP651), which was sequenced twice, to the previously used dataset. The sequencing library was prepared using the Rapid Barcoding Kit 24 V14, and sequencing was performed on the MinION Mk1B platform (Oxford Nanopore Technologies, Oxford, UK) with R10.4.1 flow cells. Then, both replicates were processed the same way as the other samples in the dataset. The obtained methylation profiles were analyzed using the PanMethylomeMiner with the same settings as in the previous case. The two replicates exhibited minimal differences (approximately 1.4 %) and clustered together in both 5mC and 6mA heatmaps (see Supplementary Fig. S4 A, B). In the case of 4mC, no significant clusters were observed, which is probably due to the low number of this type’s methylation sites (see Supplementary Fig. S4 C). The obtained results thus support the robustness and reliability of the downstream analysis performed by MethylomeMiner.

### 3.5 Computational performance

MethylomeMiner and PanMethylomeMiner were evaluated for the computational and space complexity. Execution time and memory requirements were measured on a PC equipped with Intel Xeon E-2388 G @3.20 GHz (4 cores, 2 threads per core) and 16 GB of RAM. Each measurement was performed five times, and the presented values are their averages.

MethylomeMiner was executed separately for all 20 genomes. On average, the runtime per genome was 31.28 s, with memory consumption of 7.70 GB. The most time-consuming part of MethylomeMiner is the sorting of filtered methylations into coding and non-coding groups, which, in the worst-case scenario, requires *O*(*nm*) time, where *n* is the number of filtered base modifications in the bedMethyl file and *m* is the number of coding features in the genome annotation. For the testing dataset, the average number of filtered modifications was 61,560, and the average number of CDS in annotations was 5268.

The runtime of PanMethylomeMiner depends on whether MethylomeMiner must be executed for the input files and on whether the user requests the generation of heatmaps. In the best-case scenario, when all files have already been processed by MethylomeMiner and no heatmap is requested, the panmethylome matrix is obtained in 1.52 s, with the time complexity of *O*(*mop*), where *m* represents the number of features in the extended annotation with MethylomeMiner results, *o* is the count of lines in the Roary gene_presence_absence.csv file, and *p* is the number of genomes in the pangenome analysis. For the testing dataset of 20 extended annotations (each containing on average 5268 coding features), the Roary file contained 9088 genes. In the case of a heatmap request, the runtime increased to 18.16 s to create a “compact” heatmap and to 151.07 s for a “full” heatmap.

## 4 Conclusion

This paper presents MethylomeMiner, a Python-based tool to analyze DNA methylation data obtained from nanopore sequencing. The tool works with bedMethyl files produced by Dorado and Modkit. The first module, MethylomeMiner, filters methylation sites based on coverage and the methylation rate. If an annotation file is provided, it links methylation sites to genomic features, indicating whether they are within genes or intergenic regions, and reports the surrounding context, such as neighboring genes. The second part of the tool, PanMethylomeMiner, integrates the analysis across multiple genomes. The module utilizes the results from a pangenome analysis obtained by Roary and combines methylation information for all genes in the dataset. This enables methylation profiles across strains to be compared, conserved and variable patterns to be identified, and highlights genes methylated in most genomes or only in a few. In addition, PanMethylomeMiner can produce summary tables and heatmaps that help visualize similarities and differences in methylation across the population.

Although the pangenome-level analysis in MethylomeMiner currently focuses on protein-coding regions, the tool also provides information on methylation positions in non-coding parts of the genome. Due to their variability and lack of uniform annotation, these regions are difficult to compare between strains. However, users can explore them independently and attempt to identify potential regulatory elements.

The tool’s functionality was demonstrated on a dataset of 20 *K. pneumoniae* genomes. The analysis showed that 6mA was the most common modification (63.4 %), followed by 5mC (36.4 %), while 4mC occurred only rarely (0.2 %). Furthermore, it was found that methylation occurs more frequently in non-coding regions, with 55 % of 4mC, 54 % of 5mC and 52 % of 6mA sites located outside coding sequences. On average, coding genes carried only a few methylation sites. However, two hypermethylated genes were observed in the analyzed dataset, with methylation sites exceeding 50: a chromosomal hypothetical protein observed in several genomes and a plasmid-encoded hypothetical protein detected in only one genome. Both genes were searched using BLASTp and showed high homology with phage-related proteins, suggesting that these highly methylated regions may be of phage origin. Pangenome-wide analysis revealed two similar genome clusters for both 5mC and 6mA, indicating that these modifications share related methylation patterns. These clusters did not correspond to sequence types, suggesting that other factors shape methylation profiles. In addition, for 5mC and 6mA, we identified gene sets methylated in at least 95 % of strains. Among them, three loci were consistently methylated across all genomes: *traI* for both 5mC and 6mA, and *nirB* and the S/N-oxide reductase for 6mA.

MethylomeMiner provides a user-friendly solution to process large-scale methylation data from nanopore sequencing and enables consistent, reproducible bacterial methylome studies. Thanks to its modular design, individual workflow parts can also be run separately, making the tool flexible and easy to adapt to different analyses. The obtained results can then be linked with Clusters of Orthologous Genes (COG) categories to identify the functional groups most often affected by methylation. When using whole-genome sequencing data, it is possible to compare methylation patterns between chromosomes and plasmids or to focus on specific genes of interest. The outputs can also be used in subsequent studies. For example, RNA sequencing can be applied to test whether genes identified as methylated also show changes in expression. The data can be combined with resistance or virulence profiles to identify possible links to pathogenicity. Population-level analysis also enables tracking methylation differences across strains, which could be helpful for epidemiological or clinical research.

## CRediT authorship contribution statement

**Marketa Jakubickova:** Conceptualization, Methodology, Software, Investigation, Data curation, Writing – original draft, Writing – review & editing, Supervision. **Katerina Sabatova:** Writing – original draft, Software, Data curation. **Michaela Zbudilova:** Methodology, Conceptualization. **Matej Bezdicek:** Resources, Investigation, Writing – review & editing, Data curation. **Martina Lengerova:** Writing – review & editing, Supervision, Resources. **Helena Vitkova:** Writing – review & editing, Supervision, Funding acquisition, Conceptualization.

## Funding

This work was supported by a grant project from the Czech Science Foundation [GA23-05845S] and Ministry of Health, Czech Republic - conceptual development of research organization (FNBr, 65269705).

## Declaration of competing interest

The authors declare that they have no known competing financial interests or personal relationships that could have appeared to influence the work reported in this paper.

## Data Availability

The software MethylomeMiner is openly accessible on GitHub (https://github.com/BioSys-BUT/MethylomeMiner). Data used in this study (assembled genomes, methylation tables, pangenome results, MethylomeMiner outputs) are available in the Zenodo repository (https://zenodo.org/records/16942046, [26]). All sequencing data used in this study are publicly available in the NCBI Sequence Read Archive (SRA) under accession numbers SRR35233883–SRR35233902, within BioProject PRJNA675431.
